# Emotional Situation of Children and Adolescents during the COVID-19 Pandemic in Germany: Results from the COVID-19 Snapshot Monitoring Study (COSMO)

**DOI:** 10.3390/ijerph19052698

**Published:** 2022-02-25

**Authors:** Chiara Rathgeb, Hannah Schillok, Stephan Voss, Michaela Coenen, Gerd Schulte-Körne, Christina Merkel, Sarah Eitze, Caroline Jung-Sievers, on behalf of the COSMO Study Team

**Affiliations:** 1 Institute for Medical Information Processing, Biometry, and Epidemiology—IBE, Chair of Public Health and Health Services Research, LMU Munich, Elisabeth-Winterhalter-Weg 6, 81377 Munich, Germany; chiararathgeb@outlook.de (C.R.); hannah.schillok@ibe.med.uni-muenchen.de (H.S.); svoss@ibe.med.uni-muenchen.de (S.V.); coenen@ibe.med.uni-muenchen.de (M.C.); 2 Pettenkofer School of Public Health, Elisabeth-Winterhalter-Weg 6, 81377 Munich, Germany; 3 Klinik und Poliklinik für Kinder- und Jugendpsychiatrie, Psychosomatik und Psychotherapie, Klinikum der Universität München, 80336 Munich, Germany; gerd.schulte-koerne@med.uni-muenchen.de; 4 Federal Centre for Health Education (BZgA), Maarweg 149-161, 50825 Cologne, Germany; christina.merkel@bzga.de; 5 COSMO-Study Group, Faculty of Philosophy, University of Erfurt, 99089 Erfurt, Germany; sarah.eitze@uni-erfurt.de

**Keywords:** COVID-19, mental health, emotional situation, public health, children and adolescents, SDQ

## Abstract

The COVID-19 pandemic led to numerous restrictions in daily life that had a significant impact on the well-being and mental health of the population. Among others, children and adolescents were particularly affected, being a vulnerable group at risk. The aim of this study was to assess the emotional situation of children and adolescents during different phases of the pandemic and to identify modifying factors. Data from the serial cross-sectional COVID-19 Snapshot Monitoring (COSMO) survey in Germany were used for this study. The survey waves 12 (19th/20th May 2020) and 21 (15th/16th September 2020) were investigated as examples of two different pandemic phases. The psychosocial and emotional situation and well-being of children were measured with the emotional subscale of the Strengths and Difficulties Questionnaire (SDQ) assessed by parents. Descriptive analyses and logistic regressions were calculated. In total, a third of the participating parents in wave 12 and in wave 21 reported having children and adolescents with emotional symptoms. Especially children with younger parents seemed to be more affected by emotional symptoms. Sociodemographic aspects, such as household language, showed a significant association with reported emotional symptoms in children (Wave 12: OR = 2.22; 95% CI: 1.20–4.09). Reported prevalences of emotional symptoms in children did not differ between the pandemic phases. In conclusion, the pandemic had negative influences on the emotional symptoms of children and adolescents in COVID-19 pandemic waves in 2020, indicating a forecasted reoccurrence and need for preventive measures for upcoming waves and other pandemics in the future.

## 1. Introduction

Following the global spread of the novel coronavirus (SARS-CoV-2) since the end of 2019, countries worldwide have imposed non-pharmaceutical interventions (NPIs), such as contact restrictions and closures of schools and daycare centers, as mitigation measures to contain the virus [1,2].

These measures may lead to a wide range of stressors for the young, such as loss of everyday structure; reduction of social contacts and possibilities for play, physical activities and hobbies; boredom; excessive media consumption; and loss of educational opportunities, as well as higher risks of family conflicts. Studies in Germany, but also in other countries, have confirmed that children and adolescents felt burdened by the containment strategies of the first SARS-CoV-2 waves, resulting in worsened relationships with friends, more perceived exhaustion and emotional burden through school and distance learning, and ultimately reduced quality of life [3,4,5,6]. A meta-analysis of international studies revealed that pooled estimates for depressive and anxiety symptoms were about twice as high as in prepandemic times [7].

Despite these massive curtailments experienced by children, this population group has received disproportional attention in research and prevention efforts under the current pandemic situation [8,9]. Although current strategies aim at keeping schools open as far as possible and promote health protection and promotion efforts, further restrictions (also depending on the status of vaccination recommendations) will accompany children’s everyday lives [10].

Since a child’s response and coping strategy to a crisis is determined by several factors, such as preexisting (mental) health status, socioeconomic status of the family and cultural background, it can be expected that certain subgroups will be more affected than others [4,11]. With this study, we aimed to add to the limited evidence base on the psychosocial situation of children and adolescents during the COVID-19 pandemic in Germany by analyzing emotional symptom prevalences and potential determining context factors in different pandemic waves to inform public mental-health efforts in the future and to build resilience in the young. 

We hypothesized that children and adolescents are negatively affected by the COVID-19 pandemic, and, thus, more children are emotionally burdened compared to prepandemic times. Secondly, we hypothesized that there are differences between the lockdown and the relaxation phase and that children show more emotional symptoms during the lockdown phase.

## 2. Materials and Methods

### 2.1. Study Design and Sample 

The serial cross-sectional COVID-19 Snapshot Monitoring (COSMO) study in Germany collects data during the COVID-19 pandemic on a weekly and bi-weekly basis, respectively [12]. The main aim of the COSMO study is to capture the psychological situation of German adults during the COVID-19 pandemic. Among others, pandemic-related knowledge, risk perception, behavior, acceptance of measures and trust in institutions during the pandemic are assessed by using online questionnaires [12]. The project started in March 2020 with approximately n = 1000 people per wave aged 18 to 74 years. Participants were recruited by using a quota sampling strategy. They were paid by an external sample provider according to ISO standards. Each quota sample is drawn to be representative to the German population as maintained by age, gender and federal state in terms of the German census [12,13,14].

Ethical approval was obtained by the ethics committee of the University of Erfurt. All participants took part voluntarily and had to provide informed consent. 

The COSMO study is a joint project of the University of Erfurt, the Robert Koch Institute, the Federal Centre for Health Education (BZgA), the Leibniz Centre for Psychological Information and Documentation (ZPID), the Science Media Center, the Bernhard Nocht Institute for Tropical Medicine and the Yale Institute for Global Health. In most survey waves, additional variables were collected to represent the pandemic situation and its changes. Further study details of the COSMO study are described in the study protocol, which has been adapted over time [12].

The conducted analysis was based on the data of the 12th and 21st COSMO wave. Data for the 12th wave were collected on the 19th and 20th of May 2020 during the first lockdown in Germany. Schools have been closed since the 13th of March, with the exception of children with system-relevant parents. In April, younger children in smaller groups were allowed to visit school part-time, but the rules changed frequently. After a complete lockdown in all 16 federal states of Germany and distance learning, the 12th wave marks a time of stepwise reopening of schools shortly afterward, resulting in children being taught at school in face-to-face classes again, but under very restrictive measures. Young children in daycare centers were also able to return from emergency childcare to a limited regular operation. For the 21st wave, data were collected on the 15th and 16th of September 2020, which was during the relaxation phase of the pandemic, with few restricting measures in place. It was conducted directly after the summer holidays, when face-to-face classes were held again in all federal states in Germany [15]. In addition, an increased occupancy rate in daycare centers was also recorded [16]. The time points were chosen to compare the psychological and emotional situation of children between lockdown and the relaxation phase. 

### 2.2. Variables and Measures

#### 2.2.1. Primary Outcome Variable and Exclusion Criteria

To assess our main outcome, the psychological and emotional situation of children and adolescents, the subscale of the Emotional Symptoms of the Strengths and Difficulties Questionnaire (SDQ) by Goodman was used [17]. The SDQ is a validated and internationally recognized questionnaire that yielded satisfactory results regarding the total, and to the lesser extent subscales SDQ subscales in regard to reliability, construct validity and clinical utility; it also allows researchers to identify children and adolescents at high risk for mental health problems [18,19]. The instrument evaluates positive and negative behavioral attributes of children and adolescents [20]. The SDQ was translated into German by Klasen et al. [21], using forward and backward translation, and was validated in its German version in several studies [22,23,24]. The questions here are answered by parents as a proxy report. Research has shown that SDQ scores were associated with clinical diagnoses of the young well, making the SDQ a viable instrument to screen for emotional and behavioral mental health problems among children and adolescents [25,26]. 

The SDQ has five subscales, namely Conduct Problems, Emotional Symptoms, Hyperactivity, Peer Relationships and Prosocial Behavior. Each of the five subscales consists of five questions [17], which can be answered on a 3- or 5-point Likert-Scale [27,28].

In the COSMO study, the Emotional Symptoms subscale of the SDQ was included into the survey and assessed emotional symptoms based on parents’ judgement. Surveyed parents of children aged 3 to 17 rated emotional symptoms of their children on a 3-point Likert-Scale (0 “not true”; 1 “somewhat true”; 2 “certainly true”). The Emotional Symptoms subscale included the five questions: “Often complains of headaches, stomach ache or sickness”; “Many worries/often seems worried”; “Often unhappy, down-hearted or tearful”; “Nervous or clingy in new situations, easily loses confidence”; and “Many fears, easily scared”. According to Woerner et al., a scale of 0 to 10 was generated by summing up the sub-scores of these five items, with a score range from 0 to 3 considered as “normal”, 4 referring to “borderline” and a score range from 5 to 10 as “abnormal” [29]. Children and adolescents at risk for emotional symptoms were defined as having a borderline or abnormal score. 

As parents with several children could answer the SDQ questionnaire multiple times for all their children, we saw the risk of overweighting certain family-specific context factors in our analysis and therefore decided to include only one representative child per family, the so-called “family indicator child”. Based on the frequency distribution of all children at risk for emotional symptoms on the SDQ subscale that we conducted as a pre-analysis, we decided to select the youngest child as the “family indicator child” for the emotional status. 

**Figure A1:**
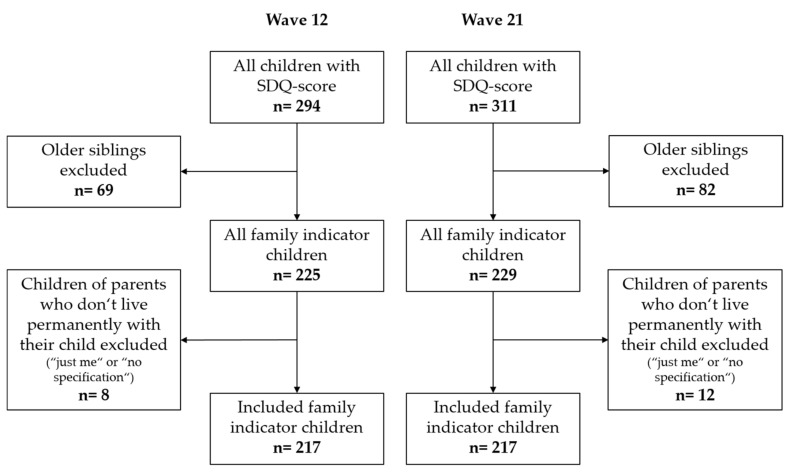
Flowchart to visualize the final study population of wave 12 and wave 21, as well as the exclusion criteria.

Children of parents were excluded from our analyses if the parent had indicated “just me” or “no specification” to describe their household size, as they may not live permanently in the same household with their children and, thus, could give less reliable information on the emotional situation of their child. As a result, we excluded 8 parents in wave 12 and 12 parents in wave 21, resulting into 217 family indicator children that were analyzed, respectively, for each wave. A flowchart to visualize the described inclusion/exclusion procedure, as well as the final study population, can be found in Appendix A, Figure A1.

#### 2.2.2. Covariates

The following covariates were analyzed: gender (male, female), age (18–29, 30–44, 45–64, ≥65 years), number of inhabitants of the municipality of residence (≤5000, 5001–20,000, 20,001–100,000, 100,001–500,000, >500,000), state of living recoded into two local German regions (East, West), education level (≤9 years of school education, ≥10 years of school education/no A-Level, ≥10 years of school education/A-Level), being self-employed (yes, no), working as a health professional (yes, no), current relationship/marriage (yes, no), household language other than German (yes, no), household size (just me, 2 persons, 3–4 persons, >4 persons, no specification), age of children (3–5, 6–9, 10–13, 14–17 years), being a single parent (yes, no) and suffering from a chronic disease (yes, no, don’t know). 

### 2.3. Statistical Analysis

For the descriptive analysis, absolute and relative frequencies were calculated for all variables for the two selected waves for the total study population and our main group of interest, parents with children between 3 and 17 years. Both populations (total study population and main group of interest) are presented.

For the evaluation of the SDQ, parents with children between 3 and 17 years were considered, since the primary outcome was only assessed in this group. For children and adolescents in different age groups, absolute and relative frequencies were given for the SDQ scores in survey waves 12 and 21. In addition, odds ratios (ORs) and 95% confidence intervals (CIs) were calculated for the two waves in this context to investigate the differences between lockdown and relaxation phase.

Univariate logistic regression models with the covariates were performed in wave 12 and 21 with the corresponding ORs and 95% CIs. The answer options “no” and “don’t know” were collapsed for the characteristics chronic disease of answering parent. The characteristic parental education was collapsed into “A-Levels” and “no A-Levels”. A multiple logistic regression model with the SDQ subscale as the dependent variable was executed, including the socioeconomic covariates gender, age of parents, parental education and household language other than German [11]. Pseudo R^2^ was calculated to assess the model fit.

All statistical analyses were conducted with the statistical program IBM SPSS Statistics 27.0 (IBM, Armonk, NY, USA). 

## 3. Results

### 3.1. Descriptive Analysis/Sample Overview

Table 1 lists all characteristics of the different variables in waves 12 and 21 in the total study population and the group of interest (parents of children aged 3 to 17). The total study population in wave 12 consisted of 972 people, out of which 225 were parents with children between 3 and 17 years of age (23.1%). In wave 21, there was a total of 1013 participants and 229 parents with children in the corresponding age group (22.6%). Parents tended to be younger, suffered less often from chronic diseases and were more often in a relationship compared to the general study population. 

### 3.2. Emotional Symptoms by the Strengths and Difficulties Questionnaire

**Figure 1:**
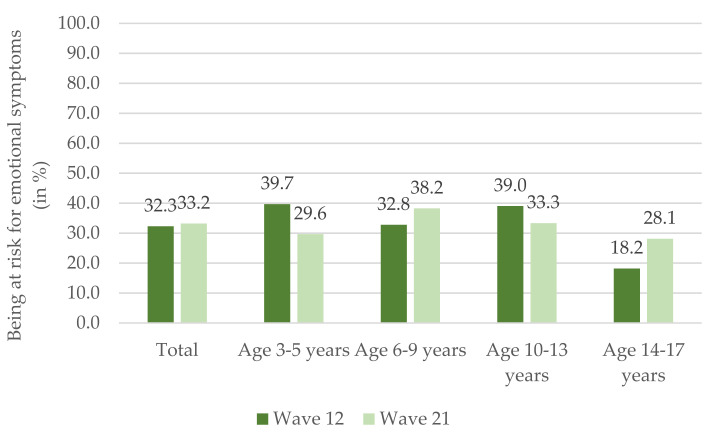
Relative frequencies of the 217 family indicator children and adolescents at risk for emotional symptoms based on the SDQ subscale in waves 12 and 21, respectively.

Figure 1 shows the relative frequencies of family indicator children and adolescents at risk for emotional symptoms based on the SDQ subscale in waves 12 and 21 (for ORs and corresponding 95% Cis, see Appendix A, Table A1). In total, 70 of 217 children and adolescents were at risk in wave 12, and 72 of 217 were in wave 21. In wave 12, most children and adolescents at risk were in the age group 3–5 years (39.7%) and 10–13 years (39.0%). In wave 21, the age group 6–9 years had the highest number of children at risk (38.2%). 

### 3.3. Univariate Analysis

Table 2 shows the absolute and relative frequencies of parents with family indicator children at risk for emotional symptoms compared to those without risk. In wave 12, the odds of having a family indicator child at risk for emotional symptoms is significantly lower for parents aged 45 to 64 years (OR = 0.21; 95% CI: 0.08–0.53) than for parents in the 18-to-29 age group. Parents with adolescents aged 14 to 17 years are also significantly less likely to have a family indicator child at risk (OR = 0.34; 95% CI: 0.14–0.79). Wave 12 also shows that parents with a household language other than German have a significantly higher probability of having a family indicator child at risk (OR = 2.22; 95% CI: 1.20–4.09). In wave 21, the results show that, in households with three or four people, the chance of having a family indicator child at risk is significantly lower (OR = 0.37; 95% CI: 0.15–0.92).

### 3.4. Multivariate Logistic Regression

The multivariate logistic regression model, including the variables gender, age of parent, parental education and household language other than German, is presented in Table 3. In wave 12, the parental age group 45 to 64 years (OR = 0.23; 95% CI: 0.09–0.62; standard error (SE): 0.50) showed a significant lower association of having a family indicator child at risk for emotional symptoms based on the SDQ subscale, and a household language other than German (OR = 2.03; 95% CI: 1.06–3.91; SE: 0.33) showed a higher association. In wave 21, none of the variables had a significant influence. The explanatory power of the model was medium in wave 12 (Pseudo-R^2^ = 0.1288) and low in wave 21 (Pseudo-R^2^ = 0.0398).

## 4. Discussion

In this study, we investigated the emotional symptoms of children and adolescents reported by their parents during the COVID-19 pandemic. Our main results showed that, in both waves (wave 12 “closely after lockdown” and wave 21 “relaxation phase” in 2020), about one-third of the investigated children were at risk for emotional symptoms based on the SDQ subscale. Possible predictors, at least in the first wave, were younger age of the parent and a household language other than German.

Comparing times of stricter pandemic mitigation measures (lockdown; wave 12) and periods of relaxation (wave 21), we could not detect any significant differences in terms of emotional symptoms within the family indicator children. Thus, our results suggest that emotional burden remains elevated in children even during relaxation phases of the pandemic. The second hypothesis, stating that differences between the pandemic phases occur, could not be confirmed.

The results of our research are in line with German studies reporting mental health problems in children during the COVID-19 pandemic [11] and a deterioration in mental health among the young during the COVID-19 pandemic internationally [30,31,32,33,34,35]. Before the pandemic, a meta-analysis from 2012 reported that about one in five children were at risk for mental health problems [36]. In the German BELLA cohort study, 17.2% of children and adolescents aged 3 to 17 years showed evidence of mental health problems, measured by the SDQ in 2017 [37]. The first hypothesis, stating that children are more emotionally stressed during the pandemic than before, could be confirmed. Regarding predicting context factors, published evidence has shown that restrictive pandemic measures during the COVID-19 pandemic affected people under 30 more severely than older ones, specifically impacting the mental health of young parents and children. Older children, however, seemed to be less negatively influenced than younger children [4,37,38,39]. Naturally, the parental age is also strongly correlated with the age of the children, so that older parents tend to have older children, which seemed to be a protective factor for children’s mental health. One explanation for this could be that younger children rely on more parental care, also in the context of distance learning. Older children with presumably older parents are probably more settled in their daily lives and are more independent.

In regard to household language other than German, which was used as a proxy for a migration background, our findings are also consistent with results from similar studies. Existing works in the literature have shown that a migration background correlates with higher stress levels during the COVID-19 pandemic and that the risk of psychological impairments is likewise increased [4,11]. As the pandemic progresses further, existing deficiencies in the healthcare system might exacerbate this effect, due to high access barriers to health information or adequate healthcare [4]. Explanations could be existing language barriers, socioeconomic aspects and differences, etc.

### Strengths and Limitations

One advantage of the COSMO study is that it allows an overview of the current pandemic situation in regard to a broad range of psychosocial factors in the German general population. The study population represents the general population in terms of gender, age and place of residence, and this is why conclusions can be generalized to the German population. Some of the topics are repeatedly asked in different waves, thus allowing researchers to analyze changes over time.

On the other hand, there are some shortcomings, as well. The study populations in the COSMO study are usually large, but particular extremes or subgroups might be underrepresented. As the snapshot gives an overview of different topics, deeper insights on a single topic often cannot be made. For instance, only one SDQ subscale was used in COSMO (subscale on Emotional Symptoms). This subscale gives an indication of emotional problems but does not allow screening of mental disorders in children and adolescents. Moreover, comparisons to other studies are limited, as mostly the SDQ with its five subscales is used in other research contexts.

Regarding the population, it should be noted that parents with children under 3 years of age were not included in the analysis, due to a lack of data for the primary outcome, although these children may also suffer from emotional problems, too. In addition, parents’ judgement might not necessarily reflect the actual situation of the children themselves [37].

In addition, we could not take into account information on parents’ and caregivers’ mental health problems, since this information was not evaluated in COSMO at the respective time points.

Moreover, the data were self-reported; thus, they are susceptible to recall bias and bias of social desirability. The fact that we could not see the same predicting factors in all waves may stem from a more complex interaction of multiple socioeconomic and family-related context factors.

Finally, in our analyses and more specifically in the multivariate regression analyses, we included covariates based on clinical relevance, previous studies/literature/evidence and availability in COSMO. However, we were limited in the selection of covariates to the availability of data assessed in the COMSO survey waves. Further research should take into account covariates such as parental mental health status, as well as more details on the biopsychosocial situation of children.

## 5. Conclusions

This study provides a snapshot of the emotional situation of children and adolescents in Germany during the earlier COVID-19 pandemic phases. Given the comparatively high prevalence of children and adolescents at high risk for emotional symptoms, effective strategies for mental-health promotion and prevention for the upcoming waves and pandemics in the young are needed.

## Figures and Tables

**Table 1:** Sample characteristics.

Sociodemographic Characteristics	Wave 12 (19th/20th May 2020)				Wave 21 (15th/16th September 2020)			
	Total *n* = 972		Parents of children 3–17 Years *n* = 225		Total *n* = 1013		Parents of children 3–17 Years *n* = 229	
	N	%	*n*	%	N	%	*n*	%
Total	972	100	225	100	1013	100	229	100
Gender of answering parent								
Male	477	49.1	109	48.4	506	50.0	109	47.6
Female	495	50.9	116	51.6	507	50.0	120	52.4
Age of answering parent								
18–29	188	19.3	27	12.0	191	18.9	18	7.9
30–44	310	31.9	118	52.4	304	30.0	138	60.3
45–64	354	36.4	77	34.2	366	36.1	71	31.0
≥65	120	12.3	3	1.3	152	15.0	2	0.9
Number of inhabitants of answering parent’s place of residence								
≤5000	148	15.2	33	14.7	168	16.6	42	18.3
5001–20,000	211	21.7	53	23.6	203	20.0	49	21.4
20,001–100,000	241	24.8	53	23.6	267	26.4	62	27.1
100,001–500,000	183	18.8	39	17.3	190	18.8	38	16.6
>500,000	189	19.4	47	20.9	185	18.3	38	16.6
Local region of answering parent’s place of residence								
East	199	20.5	51	22.7	207	20.4	48	21.0
West	773	79.5	174	77.3	806	79.6	181	79.0
Education of answering parent								
≤9 years	105	10.8	13	5.8	121	11.9	21	9.2
≥10 years (no A-Level)	334	34.4	88	39.1	357	35.2	81	35.4
≥10 years (A-Level)	533	54.8	124	55.1	535	52.8	127	55.5
Self-employed status of answering parent								
Yes	100	10.3	27	12.0	77	7.6	19	8.3
No	872	89.7	198	88.0	936	92.4	210	91.7
Health professional occupation of answering parent								
Yes	96	9.9	34	15.1	66	6.5	20	8.7
No	876	90.1	191	84.9	947	93.5	209	91.3
Relationship/marriage of answering parent								
Yes	621	63.9	193	85.8	639	63.1	191	83.4
No	351	36.1	32	14.2	374	36.9	38	16.6
Household language other than German								
Yes	244	25.1	64	28.4	257	25.4	66	28.8
No	728	74.9	161	71.6	756	74.6	163	71.2
Household size of answering parent								
Just me	284	29.2	7	3.1	294	29.0	12	5.2
2 persons	351	36.1	20	8.9	369	36.4	22	9.6
3–4 persons	276	28.4	162	72.0	297	29.3	156	68.1
>4 persons	59	6.1	35	15.6	51	5.0	39	17.0
No specification	2	0.2	1	0.4	2	0.2	0	0.0
Age of all children of parents								
Total	x	x	294	x	x	X	311	x
3–5	x	x	64	x	x	X	55	x
6–9	x	x	77	x	x	X	87	x
10–13	x	x	72	x	x	X	107	x
14–17	x	x	81	x	x	X	62	x
Single-parent status								
Yes	x	x	35	15.6	x	X	40	17.5
No	x	x	190	84.4	x	X	189	82.5
Chronic disease of answering parent								
Yes	347	35.7	65	28.9	347	34.3	68	29.7
No	600	61.7	156	69.3	643	63.5	156	68.1
Don’t know	25	2.6	4	1.8	23	2.3	5	2.2
All children with SDQ subscale Emotional Symptoms Ratings			294				311	
Not at risk	x	x	202	x	x	X	218	x
Family indicator children at risk by SDQ subscale Emotional Symptoms (at risk/not at risk) (rated by 217 parents per wave) **			217				217	
Not at risk	x	x	147 ***	x	x	X	145 ***	X
At risk	x	x	70 ***	x	x	X	72 ***	X

Note: ** includes one family indictor child of 217 parents per wave; *** of 217 parents per wave, as 8 out of 225 parents for wave 12 and 12 out of 229 parents for wave 21 got excluded based on the information given on their household size (“just me” or “no specification”).

**Table 2:** Univariate analysis—absolute and relative frequencies and odds ratios of parents with family indicator children and adolescents at risk for emotional symptoms based on the SDQ subscale.

Characteristics	Wave 12—19th/20th May 2020 (Parents with Family Indicator Children at Risk for Emotional Symptoms *n* = 70 vs. Parents without Family Indicator Children at Risk *n* = 147)				Wave 21—15th/16th September 2020 (Parents with Family Indicator Children at Risk for Emotional Symptoms *n* = 72 vs. Parents without Family Indicator Children at Risk *n* = 145)			
	*n*	%	OR	95% CI	*n*	%	OR	95% CI
Gender of answering parent								
Male (reference)	36	51.4			29	40.3		
Female	34	48.6	0.79	[0.45–1.40]	43	59.7	1.35	[0.76–2.39]
Age of answering parent								
18–29 (reference)	15	21.4			4	5.6		
30–44	40	57.1	0.43	[0.18–1.01]	41	56.9	1.45	[0.45–4.71]
45–64	15	21.4	0.21	[0.08–0.53]	27	37.5	2.31	[0.68–7.86]
Number of inhabitants of answering parent’s place of residence								
≤5000 (reference)	8	11.4			14	19.4		
5001–20,000	15	21.4	1.29	[0.47–3.51]	13	18.1	0.66	[0.27–1.65]
20,001–100,000	19	27.1	1.78	[0.67–4.75]	20	27.8	0.97	[0.41–2.26]
100,001–500,000	12	17.1	1.38	[0.48–3.97]	12	16.7	0.86	[0.33–2.22]
>500,000	16	22.9	1.60	[0.59–4.37]	13	18.1	1.01	[0.39–2.59]
Local region of answering parent’s place of residence								
East (reference)	16	22.9			13	18.1		
West	54	77.1	0.94	[0.47–1.86]	59	81.9	1.13	[0.55–2.34]
Education of answering parent								
No A-Level (reference)	27	38.6			32	44.4		
A-Level	43	61.4	1.41	[0.79–2.52]	40	55.6	1.04	[0.59–1.84]
Self-employed status of answering parent								
No (reference)	60	85.7			68	94.4		
Yes	10	14.3	1.27	[0.55–2.95]	4	5.6	0.65	[0.20–2.10]
Health professional occupation of answering parent								
No (reference)	56	80.0			65	90.3		
Yes	14	20.0	1.79	[0.83–3.85]	7	9.7	1.19	[0.45–3.17]
Relationship/marriage of answering parent								
No (reference)	12	17.1			11	15.3		
Yes	58	82.9	0.51	[0.22–1.17]	61	84.7	0.74	[0.33–1.67]
Household language other than German								
No (reference)	42	60.0			50	69.4		
Yes	28	40.0	2.22	[1.20–4.09]	22	30.6	1.12	[0.60–2.07]
Household size of answering parent								
2 persons (reference)	9	12.9			12	16.7		
3–4 persons	49	70.0	0.53	[0.21–1.36]	48	66.7	0.37	[0.15–0.92]
>4 persons	12	17.1	0.64	[0.21–1.96]	12	16.7	0.37	[0.13–1.09]
Age of Family Indicator Children								
3–5 (reference)	25	35.7			16	22.2		
6–9	19	27.1	0.74	[0.35–1.56]	26	36.1	1.47	[0.69–3.15]
10–13	16	22.9	0.97	[0.43–2.18]	21	29.2	1.19	[0.54–2.60]
14–17	10	14.3	0.34	[0.14–0.79]	9	12.5	0.93	[0.35–2.44]
Single-parent status								
No (reference)	56	80.0			59	81.9		
Yes	14	20.0	1.50	[0.71–3.16]	13	18.1	1.01	[0.48–2.10]
Chronic disease of answering parent								
No/don’t know (reference)	48	68.6			51	70.8		
Yes	22	31.4	1.18	[0.64–2.20]	21	29.2	1.04	[0.56–1.95]

**Table 3:** Multivariate logistic regression—relative frequencies and odds ratios of parents with children and adolescents at risk for emotional symptoms.

Characteristics	Wave 12—19th/20th May 2020 (Parents with Family Indicator Children at Risk for Emotional Symptoms *n* = 70 vs. Parents with Family Indicator Children Not at Risk *n* = 147)				Wave 21—15th/16th September 2020 (Parents with Family Indicator Children at Risk for Emotional Symptoms *n* = 72 vs. Parents with Family Indicator Children Not at Risk *n* = 145)			
	%	OR	95% CI	SE	%	OR	95% CI	SE
Gender of answering parent								
Male (reference)	51.4				40.3			
Female	48.6	0.76	[0.41–1.38]	0.31	59.7	1.43	[0.79–2.60]	0.30
Age of answering parent								
18–29 (reference)	21.4				5.6			
30–44	57.1	0.52	[0.21–1.27]	0.46	56.9	1.36	[0.41–4.46]	0.61
45–64	21.4	0.23	[0.09–0.62]	0.50	37.5	2.31	[0.67–7.95]	0.63
Education of answering parent								
No A-Level (reference)	38.6				44.4			
A-Level	61.4	1.51	[0.82–2.80]	0.31	55.6	1.16	[0.64–2.09]	0.30
Household language other than German								
No (reference)	60.0				69.4			
Yes	40.0	2.03	[1.06–3.91]	0.33	30.6	1.12	[0.60–2.10]	0.32
Pseudo-R²	0.1288				0.0398			

## Data Availability

For further information, see https://projekte.uni-erfurt.de/cosmo2020/web/ (accessed on 20 December 2021).

## Appendix A. Statistical Analysis

**Table A1:** Score of family indicator children and adolescents (*n* = 217 per wave) for emotional symptoms—odds ratios.

Characteristics	OR (Wave 12 vs. Wave 21) Reference Wave 12	95% CI
Total	1.04	[0.70–1.56]
Children 3–5 years	0.64	[0.30–1.38]
Children 6–9 years	1.12	[0.58–2.16]
Children 10–13 years	0.83	[0.43–1.58]
Children 14–17 years	1.49	[0.66–3.36]
